# Time to Cancer Screening Completion Following an SMS Reminder Intervention in a Large Federally Qualified Health Center Network: Secondary Data Analysis

**DOI:** 10.2196/93912

**Published:** 2026-09-03

**Authors:** Tonghui Xu, Summer Chavez, Ben King, Gonzalo Ramirez-Pulido, Chinedum O Ojinnaka, Daniel Osayi, Valery Kounga, Omolola E Adepoju

**Affiliations:** 1 Humana Integrated Health Systems Sciences Institute, University of Houston, 5055 Medical Cir, Houston, TX, 77024, United States, 1 713-743-9706; 2 Tilman J. Fertitta Family College of MedicineX, University of Houston, Houston, TX, United States; 3 Richard M. Fairbanks School of Public Health, Indiana University, Indianapolis, IN, United States

**Keywords:** cancer screening, SMS text messaging, transportation, social determinants of health, mammography, FIT, HPV testing, PAP testing

## Abstract

**Background:**

Delays in completing cancer screening diminish the preventive benefits of early detection, particularly among women receiving care in Federally Qualified Health Centers (FQHCs). Although many patients receive SMS reminders and complete screening, less is known about how quickly they complete testing or which patient-level and structural factors are associated with delays.

**Objective:**

This study examined factors associated with time to cancer screening completion among women aged 50 years or older who received SMS reminders and completed screening across a large FQHC network in Texas. The study also compared time to completion across three cancer screening tests: human papillomavirus (HPV) or the Papanicolaou test (hereinafter “Pap test”), mammography, and the fecal immunochemical test (FIT) or Cologuard screening.

**Methods:**

We conducted a secondary data analysis using electronic health record (EHR) data from a 56-clinic FQHC network in Texas. The initial cohort included 1803 women aged 50 years or older who (1) were overdue for HPV or Pap testing, mammography, or FIT or Cologuard screening, and (2) received at least three SMS reminders. Of those, 551 completed the screening and constituted the analytic cohort for this study. The outcome was the number of days from the initial SMS reminder to documented completion of the overdue screening test in the EHR. Kaplan-Meier methods were used to estimate time to completion by screening modality. A multivariable Cox proportional hazards model assessed associations of screening modality, sociodemographic, and clinical characteristics, and self-reported health-related social needs with the rate of screening completion.

**Results:**

Overall, 40.8% (n=212) of patients overdue for HPV or Pap testing completed their screening, while 21.1% (n=138) of those overdue for mammography and 32.1% (n=201) of those overdue for FIT or Cologuard screening completed their respective screening. Median time to completion was 72.5 (95% CI 64‐86) days for HPV or Pap screening and 52.0 days for both mammography (95% CI 43‐64) and FIT or Cologuard screening (95% CI 52‐53). In the adjusted model, screening completion was faster for FIT or Cologuard screening (hazard ratio [HR] 1.65, 95% CI 1.34‐2.05) and mammography (HR 1.41, 95% CI 1.11‐1.78) than for HPV or Pap screening. Patient-reported transportation limitation was associated with slower screening completion (HR 0.74, 95% CI 0.55‐0.99).

**Conclusions:**

These findings demonstrate meaningful variation in both the completion and timeliness of overdue cancer screening across screening modalities. Although HPV or Pap testing had the highest overall completion rate, time to completion was significantly shorter for mammography and FIT or Cologuard screening. The association between transportation limitations and delayed screening further underscores the influence of access-related barriers on timely preventive care. This suggests that efforts to improve cancer screening should extend beyond patient outreach to incorporate modality-specific strategies and interventions that address structural barriers to screening completion.

## Introduction

Routine cancer screening is a significant contributor to the persistent decline in cancer mortality [1]; Federally Qualified Health Centers (FQHCs) provide preventive and primary care to almost 30 million individuals in the United States; yet, preventable cancer screening rates remain significantly lower among patients who receive care in FQHCs than among the general population [2-4]. Past studies comparing rates of cancer screenings have found persistent disparities in FQHC populations, regardless of cancer type including breast (FQHC: 45.4% vs general population: 78.2%), colorectal (FQHC: 40.2% vs general population: 72.3%), or cervical cancer (FQHC: 51.0% vs general population: 82.9%) [3,4]. FQHCs are required to report standardized quality measures, including breast, cervical, and colorectal cancer screening aligned with US Preventive Services Task Force (USPSTF) recommendations, and higher performance on these measures is associated with better preventive care delivery and progress toward national screening goals [5-7]. A growing body of evidence, including past research by our team, suggests that SMS text reminders for FQHC patients overdue for their cancer screenings may be an effective strategy toward reducing this disparity in preventive care [8-10].

Meta-analytic and clinical trial evidence support SMS reminders as a useful nudge that increases screening uptake for cervical, breast, and CRC screening, though absolute effects vary (often ~2‐5 percentage points in mammography trials and larger in targeted fecal immunochemical test [FIT] programs) [6,7,10-18]. Studies also vary in the type of cancer, addition of phone calls or mailed notices, and message content [6,7,10-18]. Most previous studies have evaluated whether SMS-based interventions increase cancer screening completion. However, less attention has been paid to the timeliness of completion among patients who ultimately complete screening. One prior study in FQHC settings reported median intervals of 72 and 83 days from an abnormal FIT result to follow-up colonoscopy [19], suggesting that substantial delays may persist even among patients who ultimately complete the recommended follow-up. Patients receiving care at FQHCs often face socioeconomic and structural barriers [20] and longer times to screening completion, which may reduce opportunities for timely detection and follow-up and diminish the potential benefits of cancer screening.

Populations served by FQHCs are especially impacted by social determinants of health (SDOH), with common unmet social needs including transportation, food insecurity, and social support contributing to lower cancer screening rates. Individuals who lack reliable transportation are less likely to obtain guideline recommended cancer screenings, with one study reporting 41% lower odds of mammography adherence, independent of other SDOH [21,22]. However, it is unclear to what extent and which specific SDOH may facilitate or act as a barrier to SMS text reminder implementation in safety net populations. Previous studies have shown that time to screening completion varies across patient groups. For example, Vives et al [23] found that used FIT kits were returned sooner by women, older adults, individuals with higher deprivation scores, and those with prior screening experience. Similarly, a recent study found that younger rural adults took longer to return FIT kits, whereas women returned them sooner than men [24]. These findings demonstrate the value of examining time to completion in addition to overall screening completion.

Building on this prior body of evidence, this study examined factors associated with time to cancer screening completion among women aged 50 years or older who received SMS reminders and completed screening within a large FQHC network in Texas. The screening tests included human papillomavirus (HPV) or Papanicolaou testing (hereinafter “Pap testing”), mammography, the FIT, and Cologuard screening. Studies of this nature highlight how screening modality, socioeconomic factors, and health-related social needs may influence delays in completing recommended preventive care.

## Methods

### Data Source

Secondary electronic health record (EHR) data were obtained from a quality improvement (QI) intervention that examined the impact of SMS text message reminders on cancer screening test completion. The QI intervention was conducted at a large FQHC network in Texas. Because age ranges differ across the three cancer screening modalities, we restricted the analytic sample to women aged 50 years or older who had received at least three reminder SMS messages (three consecutive weeks) to complete any of the following overdue cancer screening tests: (1) HPV or Pap test, (2) mammography, or (3) FIT or Cologuard screening. For FIT and Cologuard screening, the FQHC provided home delivery of the test kits.

### Intervention Design and Setting

The QI initiative used SMS messages to contact patients identified through the EHR as overdue for breast, cervical, or colorectal cancer screening. The present study examined the time from the first SMS reminder to EHR-documented completion of the overdue screening test and factors associated with the rate of completion. The rolling intervention was implemented from May 2023 through June 2024. The unit of analysis was the screening episode. Approximately 5% of patients were eligible for two screening modalities and could therefore contribute one observation to each relevant modality-specific analysis.

### Participants and Eligibility

Patients were eligible for our analysis if they were (1) female and aged 50 years or older at the time of the first SMS reminder, (2) overdue for HPV or Pap testing, mammography, or FIT or Cologuard screening as of May 2023, (3) received at least three SMS reminders related to the overdue test, and (4) subsequently completed the corresponding screening test. Patients whose screening had been completed before the initial SMS reminder were excluded. Because this study focused on the timeliness of screening completion among patients who received the intervention messages and subsequently completed screening, we excluded patients in the control group, those who did not complete screening, and those who opted out.

### The QI Intervention

During the intervention period, the FQHCs implemented an SMS text messaging reminder initiative for eligible female patients who were overdue for cancer screening. SMS messages were sent in English and Spanish and tailored to the cancer screening test for which the patient was overdue. The intervention messages were tailored to each patient’s cancer test type: colorectal, breast, or cervical cancer screening. Patients received between 3 and 16 messages during the intervention period. The timing and frequency of messages varied across weeks and intervention pathways. Messages were generally sent between 9 AM and 11:30 AM Central Time, most often on Tuesdays through Fridays. Most messages were interactive, allowing patients to respond directly to the SMS messages or click embedded links to watch a short video, or to access additional information and scheduling resources. Some messages provided information about cancer screening tests, some included links to schedule cancer screening appointments, and some provided educational links about the benefits of screening and the risks of not completing screening.

Examples of the breast messages included, “Dense breasts have more fibrous tissue than fat. It is harder to spot cancer in dense breasts on a mammogram. If your mammogram shows dense breasts, you might be asked to get an ultrasound or MRI for a better view. Do it! Learn more: https://msg.care/8SSoY.”

Another example was, “Hi %{patient_first_name}! %{provider_short_name} reminds you that you are due for a mammogram This is an important screening test for breast cancer. Call us at ###-###-#### to schedule.”

### Outcome and Follow-Up

The event was completion of the screening test for which the patient was overdue, as documented in the EHR. Time to completion was calculated as the number of days from the date of the first SMS reminder to the date of documented test completion. Patients without documented screening completion or who opted out were excluded from this analysis. The latest EHR observation date available in our dataset was July 2024, and the maximum observed time to completion was 249 days. Information on death, transfer of care, and screening completed outside the FQHC network was not consistently available and therefore could not be incorporated.

### Covariates

Covariates were selected a priori based on their plausible relationships with access to, and completion of, preventive screening. EHR-derived variables included age at the first SMS reminder, insurance status, primary language, race and ethnicity, completion of an annual wellness visit during the preceding (12 mo per calendar year), BMI category, and documented histories of depression, hypertension, diabetes, and family history of cancer.

Age was categorized as 50‐54, 55‐59, 60‐64, or 65 years or older. Insurance was categorized as uninsured, private insurance, Medicaid, or Medicare. Primary language was categorized as English, or Spanish or another language. Race and ethnicity were categorized as non-Hispanic White, non-Hispanic Black, Hispanic, or another race or multiple races and ethnicities. Overweight or obesity was defined as a BMI of ≥25 kg/m². Health-related social needs (HRSN) were obtained from patients’ responses to the program’s routine screening questionnaire and included social isolation, food insecurity, housing insecurity, and transportation limitations. Each measure was coded as positive or negative. The most recent response recorded before the first SMS reminder was used.

### Missing Data

EHR-derived demographic and clinical covariates were complete in the source data. Missingness was present in the self-reported HRSN measures: 50% for food insecurity, 50% for housing insecurity, 44% for transportation limitations, and 43% for social isolation. No values were imputed. We compared demographic characteristics between observations included in and excluded from the complete-case analysis and found no substantial differences between the groups.

### Statistical Analysis

We first summarized the characteristics of the study population using descriptive statistics. The time-to-event outcome, defined as the number of days from the start of the intervention to screening completion, was visualized using Kaplan-Meier curves. We plotted stratified curves for each cancer screening test type to visually compare completion trends. The proportional hazards assumption was assessed using Schoenfeld residuals, and no violations were detected (all *P* values were >.05). Subsequently, a Cox proportional hazards model was applied to quantify the associations between time to screening completion and the independent variables among patients who completed screening [25]. Hazard ratios (HRs) and their 95% CIs were derived by exponentiating the model coefficients. We also computed multicollinearity using variance inflation factors (VIFs) to ensure model stability. All analyses were performed using R (version 4.4.2) in RStudio (Posit, PBC), using the survival package. All tests were 2-sided with a significance level of *P*<.05.

### Ethical Considerations

The University of Houston’s Institutional Review Board reviewed and approved this secondary analysis of EHR data (STUDY00004081). The board approved waivers of informed consent because the study used existing clinical and QI data and involved no additional participant contact. The analytic dataset was deidentified before sharing with the research team for analysis and was stored on access-controlled institutional systems. Patients received no compensation for their inclusion in this secondary data analysis. This manuscript was prepared in accordance with STROBE (Strengthening the Reporting of Observational Studies in Epidemiology) guidelines for cohort studies.

## Results

Table S1 in Multimedia Appendix 1 shows screening completion rates overall and by screening type. The total sample of patients who received SMS reminders included 1803 individuals. In the HPV or Pap test group, 212 (40.38%) patients completed screening, while 313 (59.62%) did not complete screening. In the mammography group, 138 (21.10%) patients completed screening, while 516 (78.90%) did not complete screening. In the FIT or Cologuard screening group, 201 (32.21%) patients completed screening, while 423 (67.79%) did not complete screening. Overall, 551 (30.56%) patients completed screening, and 1252 (69.44%) did not complete screening. Table 1 presents the descriptive statistics of the study sample. Among participants who completed a cancer screening test, 38.48% (n=212) were in the HPV or Pap test group, 25.05% (n=138) in the mammogram group, and 36.47% (n=201) in the FIT or Cologuard screening group. Regarding age group, 26.32% (n=145) of participants who completed a test were aged 50‐54 years, 26.68% (n=147) were aged 55‐59 years, 24.32% (n=134) were aged 60‐64 years, and 22.68% (n=125) were aged 65 years or older. The proportion of participants whose primary language was English was 53.36% (n=294). In terms of insurance coverage, 52.63% (n=290) of patients were uninsured, 28.86% (n=159) had private insurance, 4.72% (n=26) had Medicaid, and 13.79% (n=76) had Medicare. Over half of the participants were Hispanic (n=302, 54.81%), 24.32% (n=134) were non-Hispanic White, 9.07% (n=50) were Black/African American, and 11.80% (n=65) were of other races (Asian, bi- or multiracial, and other racial categories). Regarding health-related variables, 25.41% (n=140) of participants had depressive disorder, 59.17% (n=326) had hypertension, 88.75% (n=489) had overweight or obesity, 34.48% (n=190) had diabetes, 7.80% (n=43) had a family history of cancer, and 21.78% (n=120) had a wellness visit in 2022. Additionally, 12.52% (n=69) of patients reported social isolation, 4.90% (n=27) reported having food insecurity, 13.79% (n=76) reported having housing insecurity, and 9.98% (n=55) had transportation limitations.

**Table 1.** Sociodemographic characteristics of the study sample (n=551).

Variable	Patients, n (%)
Cancer screening test	
HPV^a^ or Pap^b^ test	212 (38.48)
Mammogram	138 (25.05)
Fecal immunochemical test or Cologuard screening	201 (36.47)
Age (years)	
50‐54	145 (26.32)
55‐59	147 (26.68)
60‐64	134 (24.32)
65+	125 (22.68)
Primary language	
English	294 (53.36)
Spanish and others	257 (46.64)
Insurance type	
Uninsured	290 (52.63)
Private	159 (28.86)
Medicaid	26 (4.72)
Medicare	76 (13.79)
Race/ethnicity	
Non-Hispanic White	134 (24.32)
Black or African American	50 (9.07)
Hispanic	302 (54.81)
Others	65 (11.80)
Depressive disorder	
No	411 (74.59)
Yes	140 (25.41)
Overweight or obesity	
No	62 (11.25)
Yes	489 (88.75)
Hypertension	
No	225 (40.83)
Yes	326 (59.17)
Diabetes	
No	361 (65.52)
Yes	190 (34.48)
Family cancer history	
No	508 (92.20)
Yes	43 (7.80)
Wellness visits in the prior year	
No	431 (78.22)
Yes	120 (21.78)
Social isolation	
No	482 (87.48)
Yes	69 (12.52)
Food insecurity	
No	524 (95.11)
Yes	27 (4.90)
Housing insecurity	
No	475 (86.21)
Yes	76 (13.79)
Transportation limitation	
No	496 (90.02)
Yes	55 (9.98)

a HPV: human papillomavirus.

b Pap: Papanicolaou.

**Figure 1.**
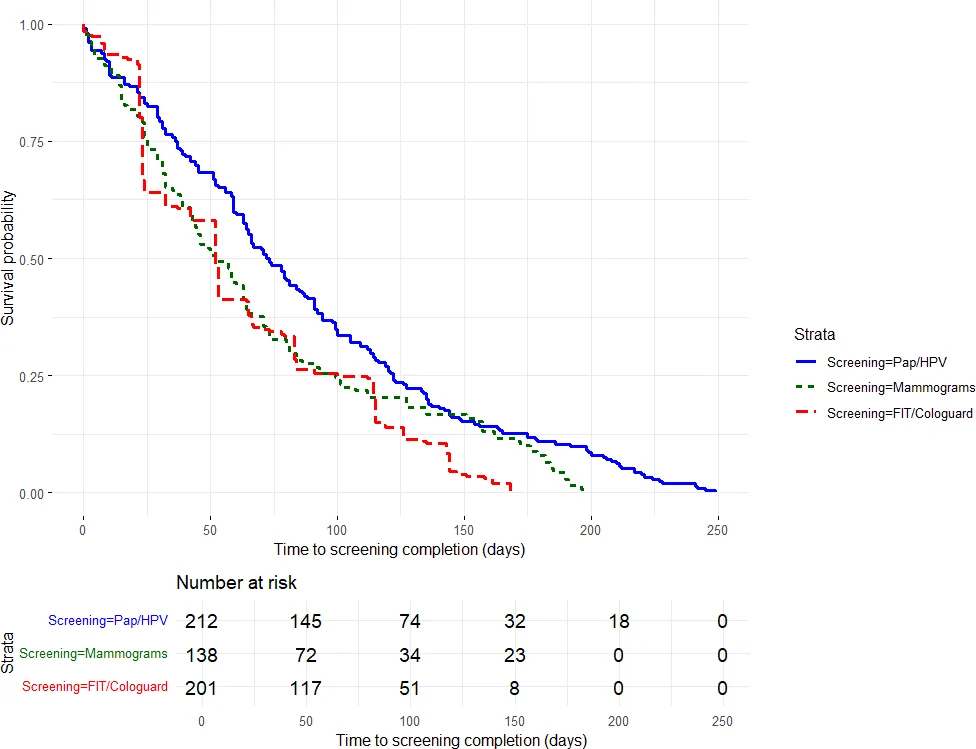
Survival probability by cancer screening type. FIT: fecal immunochemical test; HPV: human papillomavirus; Pap: Papanicolaou.

Table 2 shows the median survival times by type of cancer screening. The median survival times for the overall cohort, HPV or Pap testing, mammography, and FIT or Cologuard screening groups, were 59.0 (95% CI 53‐65), 72.5 (95% CI 64‐86), 52.0 (95% CI 43‐64), and 52.0 (95% CI 52‐53) days, respectively. Figure 1 shows Kaplan-Meier curves for time to cancer screening completion by type of cancer screening. Higher survival probabilities indicate a greater proportion of patients who had not yet completed screening at a given time point (ie, slower completion). The HPV or Pap testing cohort showed the highest survival probabilities, suggesting the slowest completion of follow-up. For the overall cohort, the completion period ranged from 0 to 248 days; for the FIT or Cologuard screening cohort, from 0 to 168 days; for the mammography cohort, from 0 to 248 days; and for the HPV or Pap testing cohort, from 0 to 248 days.

**Table 2.** Median survival times by cancer screening type.

Test	Events, n	Survival time (days), median (95% CI)
Overall	551	59.0 (53-65)
HPV^a^ or Pap^b^	212	72.5 (64-86)
Mammography	138	52.0 (43-64)
FIT^c^ or Cologuard screening	201	52.0 (52-53)

a HPV: human papillomavirus.

b Pap: Papanicolaou.

c FIT: fecal immunochemical test.

Finally, Table 3 presents the results of the Cox proportional hazards model. The model demonstrated no evidence of problematic multicollinearity (all VIFs<5). Compared to patients who were overdue for HPV or Pap screening, those in the FIT or Cologuard screening group (HR 1.65, 95% CI 1.34‐2.05; *P*<.001) and the mammography group (HR 1.41, 95% CI 1.11‐1.78; *P*<.001) had significantly shorter times to screening completion. Screening positive for transportation as a social need was associated with delayed screening completion (HR 0.74, 95% CI 0.55‐0.99; *P*=.045).

**Table 3.** Cox proportional hazards model.

Variable	HR^a^	SE	*P* value	95% CI
Cancer screening test				
HPV^b^ or Pap^c^ test	Ref^d^	—^e^	—	—
Mammogram	1.41	0.11	<.001	1.34-2.05
FIT^f^ or Cologuard screening	1.65	0.12	<.001	1.11-1.78
Age (years)				
50‐54	Ref	—	—	—
55‐59	0.85	0.12	.19	[0.67, 1.08]
60‐64	1.08	0.13	.51	0.85-1.39
65+	0.95	0.14	.74	0.75-1.32
Primary language				
English	Ref	—	—	—
Spanish and others	0.86	0.11	.19	0.69-1.08
Insurance type				
Uninsured	Ref	—	—	—
Private	0.99	0.10	.99	0.81-1.23
Medicaid	1.29	0.21	.23	0.85-1.96
Medicare	0.91	0.15	.53	0.85-1.23
Race or ethnicity				
White	Ref	—	—	—
Black or African American	0.84	0.18	.34	0.59-1.20
Hispanic	1.14	0.14	.34	0.87-1.50
Others	1.04	0.17	.82	0.75-1.45
Depressive disorder				
No	Ref	—	—	—
Yes	1.11	0.11	.34	0.90-1.37
Overweight or obesity				
No	Ref	—	—	—
Yes	0.83	0.12	.97	0.61-1.11
Hypertension				
No	Ref	—	—	—
Yes	0.85	0.10	.11	0.69-1.04
Diabetes				
No	Ref	—	—	—
Yes	1.04	0.10	.73	0.84-1.27
Wellness visits in 2022				
No	Ref	—	—	—
Yes	1.16	0.11	.19	0.93-1.43
Family member has a history of cancer				
No	Ref	—	—	—
Yes	1.09	0.17	.62	0.78-1.53
Social isolation				
No	Ref	—	—	—
Yes	0.97	0.14	.83	0.74-1.27
Food insecurity				
No	Ref	—	—	—
Yes	1.04	0.22	.99	0.66-1.53
Housing insecurity				
No	Ref	—	—	—
Yes	0.96	0.13	.79	0.73-1.26
Transportation limitation				
No	Ref	—	—	—
Yes	0.74	0.15	.045	0.55-0.99

a HR: hazard ratio.

b HPV: human papillomavirus.

c Pap: Papanicolaou.

d Ref: reference group.

e Not applicable.

f FIT: fecal immunochemical test.

## Discussion

### Principal Findings

In this study of women aged 50 years or older who completed cancer screening after receiving SMS reminders within a large FQHC network, we observed notable differences in the timeliness of screening completion across screening modalities. Compared to the HPV or Pap testing cohort, patients exhibited shorter times to screening completion for breast and colorectal cancer screening tests. Importantly, screening positive for transportation as a social need was associated with delayed screening completion, suggesting that transportation barriers may be an important factor related to timely preventive care. These findings suggest that social needs and screening modality may jointly contribute to suboptimal preventive cancer screening behaviors.

Although patients received similar intervention messages for breast, cervical, and colorectal cancer screening, the time required to complete screening differed across the three screening modalities. The finding of faster time to screening for FIT or Cologuard screening and mammography completion, compared to HPV or Pap testing, is noteworthy, and aligns with prior research elucidating variations in barriers to cancer screening across multiple sites [26,27]. Thus, our study findings may be reflective of these variations. The ability to complete FIT and Cologuard screening tests at home and return them by mail may reduce barriers such as scheduling conflicts, transportation challenges, and time constraints. The longer time to HPV or Pap testing completion may be attributable to delays in obtaining appointments with primary care providers or gynecologists, which may exceed wait times for mammography services. These differences and perceived burden may explain the shorter completion time we observed among patients assigned to the HPV or Pap testing intervention. Augmenting text message reminders with patient navigation to assist with identifying and addressing barriers to screening could reduce time to screening [28].

The observed association between transportation challenges and delayed screening completion has practical implications. Individuals may face transportation challenges due to transportation costs or difficulty navigating public transit [29,30]. This insight offers an actionable strategy for improving screening completion rates, especially among FQHC patients, and potentially reducing cancer outcomes disparities. For example, others have explored the use of mobile mammograms [31], in partnership with community stakeholders, to provide mammograms within the community and reduce transportation-related challenges. Additionally, strategies such as ride-share programs and insurance-covered transportation, which have been used to address transportation barriers to cancer treatment, may also offer an effective approach to addressing similar screening barriers [32-34]. For patients who ultimately complete screening, transportation assistance may reduce delays and facilitate more timely completion.

### Limitations and Strengths

This study is not without limitations. First, due to the focus on one FQHC network in Texas and the inclusion of only women aged 50 years and older, the generalizability of the findings may be limited. Second, because patient-level information on death, transfer of care, loss to follow-up, and the end of observation was unavailable, censoring could not be reliably defined for patients without documented screening completion. This analysis was restricted to patients with valid documented completion dates. Accordingly, the findings represent associations among screening completers and should not be interpreted as factors associated with whether screening was completed or generalized to patients who did not complete screening or opted out. The study, however, focuses on patients in an FQHC population who received SMS reminders and completed screening, allowing us to identify factors associated with faster or delayed completion among eventual completers. Third, there are other contextual factors, such as clinic-level workflows, provider recommendation patterns, and individual health beliefs, which may influence time to screening completion that are not captured in this study.

Additionally, our analysis treated each data point as independent. In reality, some individuals were likely eligible for more than one type of screening test, introducing potential correlation across observations. Still, because the study examined completion of each screening test separately, this limitation is unlikely to meaningfully affect the findings. Furthermore, HRSN variables relied on self-reported information, which may be subject to missingness or reporting bias. Patients with missing HRSN values were excluded from the analysis, which may have reduced the analytic sample size and introduced potential selection bias if those patients differed from those with complete HRSN data. Finally, due to data limitations, we were unable to control for other potential barriers to screening, such as scheduling conflicts, fear, anxiety, or preferred language other than English, which may have resulted in residual confounding.

Despite these limitations, this study has several strengths. First, our focus on patients served by FQHCs adds to the literature, given their lower screening uptake and longer time to screening completion than those of the general population. Second, our comparison of multiple screening modalities provides stakeholders with insights into differences in completion time and identifies which screening pathways may require more time or additional support for these three common cancers. Third, our inclusion of demographic, social needs, and health-related factors increases the robustness of the findings. Fourth, identifying transportation as a barrier highlights a clear and actionable target for interventions aimed at promoting timely completion of recommended cancer screening.

## Conclusions

In closing, our findings show that the same SMS reminder intervention have varied effects across cancer screening modalities, suggesting that additional demands, such as structural barriers faced by underserved patients, influence timely cancer completion. Although all patients in this study ultimately completed screening, transportation challenges were associated with delayed completion, reinforcing the need for targeted transportation support in FQHC settings. These findings suggest that SMS reminders alone may be insufficient to address structural barriers. Efforts to reduce these barriers may promote faster screening completion, enhance the effectiveness of SMS-based interventions, and help reduce inequities in cancer prevention outcomes.

## Supplementary material

10.2196/93912 Multimedia Appendix 1 Event rates by screening type (n=1803).

## Abbreviations

EHR: electronic health record
FIT: fecal immunochemical test
FQHC: Federally Qualified Health Center
HPV: human papillomavirus
HR: hazard ratio
HRSN: health-related social needs
QI: quality improvement
SDOH: social determinants of health
STROBE: Strengthening the Reporting of Observational Studies in Epidemiology
USPSTF: US Preventive Services Task Force
VIF: variance inflation factor
