# Supplementary material for: Time to Cancer Screening Completion Following an SMS Reminder Intervention in a Large Federally Qualified Health Center Network: Secondary Data Analysis
Source: JMIR Mhealth Uhealth. 2026 Sep 3;14:e93912. doi: 10.2196/93912 (PMC13540622; doi:10.2196/93912)
Supplement: Supplementary file 1 [file mhealth-v14-e93912-s001.docx]

**Multimedia Appendix 1**

**Table S1. Event Rates by Screening Type (n = 1,803)**

|  | HPV/Pap | Mammogram | FIT/ Cologuard | Overall Event | Overall exclude |
| --- | --- | --- | --- | --- | --- |
| Cancer screening test completed | 212 (40.38%) | 138 (21.10%) | 201 (32.21%) | 551 (30.56%) | -- |
| Cancer screening test not completed | 313 (59.62%) | 516 (78.90%) | 423 (67.79%) | -- | 1,252 (69.44%) |
| Total sample | 525 | 654 | 624 | -- | -- |
